# Osteoarthritis as a marker and potential contributor to cardiovascular risk: a glycocalyx–cartilage axis hypothesis

**DOI:** 10.3389/fcvm.2026.1903797

**Published:** 2026-10-05

**Authors:** Willem Wassenaar

**Affiliations:** Wellesley Therapeutics Inc., Toronto, ON, Canada

**Keywords:** articular cartilage, cardiovascular disease, endothelial glycocalyx, glycosaminoglycans, hexosamine biosynthetic pathway, N-acetylglucosamine, osteoarthritis

## Abstract

Osteoarthritis (OA) may be more than simply a reflection of aging; it could represent a symptom of underlying vascular disease processes with serious consequences for health and quality of life. The association of OA with other conditions like cardiovascular disease (CVD) suggests OA may be partially systemic and metabolic in nature. As such, this connection underscores the value of exploring the biochemical parallels between articular joints and the endothelial glycocalyx, the protective lining of blood vessels. N-acetylglucosamine may be central to this link because both articular cartilage and the endothelial glycocalyx depend on N-acetylglucosamine-containing structures. A better understanding of the pathophysiology linking OA and CVD provides the basis for the present hypothesis. Hypothesis: A reduction in available or utilized levels of N-acetylglucosamine results in a parallel degradation of articular cartilage and the endothelial glycocalyx. This would result in a shared glycan-dependent mechanism linking the pathogenesis of OA to increased cardiovascular risk. Implications: OA could serve as an early clinical marker for vascular dysfunction and potentially identify glycocalyx-targeted interventions as a new area of investigation for therapies to reduce both OA and CVD. The novelty of this research hypothesis is based on suggesting that N-acetylglucosamine-dependent glycan insufficiency is a unified mechanism connecting cartilage degeneration with vascular endothelial damage.

## Introduction

The association of osteoarthritis (OA), defined by the progressive breakdown of articular cartilage (1), with conditions such as hypertension, diabetes, and cardiovascular disease (CVD) has prompted discussions around the concept of a ‘metabolic OA phenotype’ (2). Factors like obesity, dyslipidemia, elevated fasting glucose levels, and hypertension are believed to influence the development and progression of OA. Conversely, OA itself may act as an independent metabolic risk factor for CVD (2). OA is a leading cause of disability (3), while CVD remains a leading cause of mortality (4), underscoring the clinical importance of understanding potential links between these conditions.

Several studies have identified a positive association between OA and both CVD and metabolic syndrome (2, 5). For instance, an epidemiological association was supported by the results of a large population cohort study of 18,490 participants over a 13.4-year period (2). The study demonstrated a higher risk of CVD events in patients with OA (2). Nearly one-third (31.9%) of such individuals experienced a cardiovascular event, with an even higher CVD risk observed in people with OA in the knee and hip joints (2). People with self-reported difficulty walking reported a 30% increased risk for CVD events (2). Another longitudinal study of 51,938 participants also established a link between OA and CVD risk (5). The study reported that radiographically confirmed OA was associated with an increased risk of mortality from CVD with a hazard ratio (HR) of 1.43 (95% CI 1.32–1.64) and mortality related to diabetes with a HR of 2.04 (95% CI 1.87–2.23) (5). The study also reported a direct correlation between the age of OA onset and the risk of death. Compared with participants without radiographically confirmed OA, all-cause mortality was higher in those with OA diagnosed before age 40 [HR 1.53 (95% CI 1.43–1.65)] (5). The magnitude of these relationships, particularly in younger individuals, draws attention to the possibility that OA may precede or actively contribute to systemic vascular dysfunction beyond the risk associated with sedentary behaviour from age-related pain. Recent epidemiologic studies continue to report a very strong association between OA and CVD (6). Both conditions are also associated with cardiometabolic diseases such as insulin resistance, type 2 diabetes mellitus, hypertension and dyslipidemia. These observations are consistent with the possibility that a shared biological mechanism may contribute to both joint and vascular pathology. Our hypothesis is framed as a specific mechanistic model that could operate alongside existing explanations linking OA and CVD risk.

We hypothesize that reduced availability or utilization of N-acetylglucosamine contributes to parallel degradation of articular cartilage and the endothelial glycocalyx, linking OA with increased CVD risk through a shared glycan-dependent mechanism. To our knowledge, this manuscript is the first to explicitly propose N-acetylglucosamine-dependent glycan insufficiency as a unifying mechanistic link between cartilage degeneration and endothelial glycocalyx injury in the OA–CVD association.

### Proposed mechanistic link: the glycocalyx–cartilage axis

Blood vessels are lined by carbohydrate molecules which share glycan components with cartilage (7). This blood vessel lining, called the endothelial glycocalyx, is a polymeric sugar-rich network covering the apical surface of vascular endothelial cells (7). We propose that this biochemical similarity reflects a shared dependence on N-acetylglucosamine-derived glycans, forming the basis of a unified pathophysiological mechanism linking degeneration of both cartilage and the endothelial glycocalyx. This is the central hypothesis of the manuscript and is intended as a mechanistic explanation for the epidemiological association between OA and CVD.

The purpose of Table 1 is to show that cartilage and the endothelial glycocalyx share core glycan constituents relevant to the proposed mechanism. Although the molecules in Table 1 are named differently, they share common disaccharide structures derived from glucose and its derivatives. These shared biosynthetic origins support the plausibility of a systemic mechanism affecting both cartilage and the endothelial glycocalyx and the possibility that depletion of shared carbohydrate substrates could simultaneously impair both cartilage integrity and vascular endothelial function.

**Table 1:** Glycan structures shared between the articular cartilage and the endothelial glycocalyx^a^ (8).

Molecule	Composition^b^	Shared?
Hyaluronan	N-Acetylglucosamine—Glucuronic Acid	Yes
Heparan Sulfate	N-Acetylglucosamine—Iduronic Acid	Yes
Keratan Sulfate	N-Acetylglucosamine—Galactose	Yes
Chondroitin Sulfate	N-Acetylgalactosamine—Glucuronic Acid	Yes
Dermatan Sulfate	N-Acetylgalactosamine—Iduronic Acid	Yes

a The molecules listed in the left column are shared by the endothelial glycocalyx and cartilage. The disaccharide component of these molecules (middle column) is similar but the molecular size and degree of sulfation is different in each of these two tissues. The table establishes a shared reliance on N-acetyl amino-sugar building blocks. Table 1 is a teaching tool that shows that the macromolecules in the endothelial glycocalyx and cartilage are compositionally similar. This supports the hypothesis that N-acetylglucosamine deficiency has the potential to impact both tissues; it is not offered as proof of a shared substrate deficiency, which remains the hypothesis under examination.

b N-Acetylgalactosamine and N-Acetylglucosamine and Iduronic acid and Glucuronic acid are epimers. Epimers are stereoisomers that differ in configuration at only one carbon and are interconverted. A deficiency in the hexosamine biosynthetic pathway could impact the production of both substrates.

The parallel reduction in glycocalyx thickness and cartilage carbohydrate content is consistent with, but not definitive of, a systemic deficit rather than isolated tissue-specific degeneration. In a study of normal human articular cartilage, proteoglycans made up 21% and collagen made up 79% of the collagen-carbohydrate content (1). In mild OA, the carbohydrate content is 18%, and in severe OA, the content drops to 15% (1). The human vascular glycocalyx is also reduced in disease states. In one study, the glycocalyx volume of normal controls was found to be 1.7 ± 0.6 L, and in patients with familial hypercholesterolemia, the glycocalyx volume was only 0.8 ± 0.3 L (9). In 600 healthy middle-aged subjects followed for 6 years, a thinner glycocalyx predicted more cardiovascular events (10). This pattern is consistent with the proposed hypothesis that reduced N-acetylglucosamine availability may contribute to concurrent structural decline in both cartilage and vascular glycocalyx.

### Glucosamine and cardiovascular risk: observational evidence

N-Acetylglucosamine is derived from the hexosamine biosynthetic pathway (11). When working correctly, the hexosamine biosynthetic pathway converts glucose into UDP-GlcNAc (the active form of N-acetylglucosamine), which is then involved in the synthesis of glycoproteins and glycosaminoglycans used to build cartilage and blood vessel lining (12). Therefore, if the availability of N-acetylglucosamine is reduced in joint and vascular pathology, then interventions that influence hexosamine metabolism, such as glucosamine supplements, could offer indirect evidence in support of this mechanism.

For instance, the literature already shows that the use of hexosamine-containing supplements may influence carbohydrates in both cartilage and the vascular glycocalyx, although the evidence remains indirect and based largely on surrogate outcomes (13–15). In a human OA trial, glucosamine sulfate was administered over a 3-year period (13). The group that received 1,500 mg per day of glucosamine sulfate showed no change in joint space narrowing, consistent with a possible structural benefit of oral glucosamine, although direct inference regarding cartilage carbohydrate content is limited (13). In contrast, the placebo group had progressive joint space narrowing of −0.31 mm in one study (13) and −0.19 mm in another study (14). In one trial, the impact of an oral glycocalyx-targeted treatment (sulodexide) on glycocalyx thickness was measured using side-stream darkfield imaging (15). An oral supplement containing heparan sulfate (N-acetylglucosamine—iduronic acid) and dermatan sulfate (N-acetylgalactosamine—iduronic acid) was administered to 10 participants with type 2 diabetes and 10 controls without diabetes (15). Both groups were given 200 mg of the supplement daily for 8 weeks (15). In the group of participants with type 2 diabetes, the glycocalyx increased from the baseline value of 0.64 µm to 0.93 µm (*p* < 0.05), with a non-significant change for the controls [0.78 µm to 0.88 µm (NS)] (15).

Although findings are preliminary and based on small cohorts or surrogate endpoints, research has reported that glucosamine supplementation is associated with lower cardiovascular risk in observational studies (Table 2). Despite not being mechanistic, these associations are consistent with the possibility that modulation of these pathways may influence cardiovascular outcomes in a manner aligned with the proposed hypothesis (16–19).

**Table 2:** Observational evidence linking glucosamine supplementation with reduced cardiovascular and mortality risks.

Study	Cohort Size (n)	Key Findings (CVD/ Mortality)
Pocobelli et al. (2010) (16)	77,719	Glucosamine users (≥4 days/week for ≥3 years) had lower mortality rates compared to non-users.
Bell et al. (2012) (17)	77,510	Glucosamine users (mean follow-up 6.8 years) had reduced risk of total mortality [HR 0.82 (95% CI 0.75–0.90)].
Li et al. (2020) (18)	495,077	Glucosamine use associated with lower risk of all-cause mortality [HR 0.85 (95% CI 0.82–0.89)] and CVD-related mortality [HR 0.82 (95% CI 0.74–0.90)].
Ma et al. (2019) (19)	502,616	Glucosamine use associated with reduced risk of CVD events (HR 0.85, 95% CI 0.80 to 0.90), CVD deaths [HR 0.78 (95% CI 0.70–0.87)], coronary heart disease [HR 0.82 (95% CI 0.76–0.88)] and stroke [HR 0.91 (95% CI 0.83–1.00)].

These findings, while consistent, may be influenced by healthy-user bias and do not establish a direct mechanistic role for glucosamine.

## Discussion

The observed association between OA and CVD may be partially explained by a shared dependency on N-acetylglucosamine-derived glycans. Our hypothesis provides one possible mechanism, rather than the sole explanation for the OA and CVD relationship.

The biochemical vulnerability shared by the joints and the vasculature may be rooted in the hexosamine biosynthetic pathway. The hexosamine biosynthetic pathway diverts glucose entering a cell from glycolysis toward the production of N-acetylglucosamine (11). Interruption of this pathway has been shown to impair cellular proliferation and viability, underscoring the biological importance of N-acetylglucosamine (11). Whether this reflects reduced substrate availability, altered metabolic flux, or impaired cellular utilization remains unclear and represents an important area for future investigation. Both the endothelial glycocalyx and articular cartilage are “high-wear” tissues that face constant physical stress (blood flow pressure vs. joint loading) (20–22). As a result, they require a high “repair rate,” so a slight drop in N-acetylglucosamine availability might cause these high-stress tissues to fail first.

There appears to be an inverse relationship between intracellular N-acetylglucosamine and insulin resistance. In a group of eight patients with uncontrolled diabetes mellitus and severe insulin resistance, muscle biopsies were taken before and after treatment with IV insulin for a period of 28 days (23). Before insulin treatment, intracellular N-acetylglucosamine in the form of UDP-N-acetylglucosamine was found to be 8.81 ± 1.21 nmol/g of tissue. In muscle biopsies taken after the patients became euglycemic, muscle UDP-N-acetylglucosamine rose to 12.31 ± 2.52 nmol/g (*p* < 0.005). Similarly, UDP-N-Acetylgalactosamine, the epimer of N-Acetylglucosamine, rose from 4.49 ± 0.85 to 5.89 ± 1.55 nmol/g after achieving a period of euglycemia (23). Furthermore, vascular pathology at baseline predicted structural progression of OA over the subsequent 2–10 years. This suggests that vascular pathology may be the causal pathway for OA development and progression (24). Metabolic syndrome-associated abdominal obesity, hyperglycemia and insulin resistance impair vascular function and microcirculation, reducing subchondral bone perfusion and promoting chronic inflammation, which results in cartilage degeneration (25).

Additionally, age-related incidence of OA differs between men and women. In men under 45 years of age, OA rates are higher than those found in women. However, women are impacted more after the age of 55. After the age of 60, rates in men are reported to be 9.6% and in women, 18%. Similar sex differences are found in CVD (26). Premenopausal women with higher circulating estrogen levels have lower visceral fat distribution than men. As estrogen declines, visceral fat increases (25). Increased visceral fat is associated with insulin resistance and an increase in pro-inflammatory cytokines such as TNF- a and IL-6 (27). These factors may be the reason women are protected in early life and not in advanced age.

N-acetylglucosamine may have relevance to several other chronic diseases (e.g., Alzheimer's, Parkinson's, and multiple sclerosis); however, those relationships are secondary to the cardiovascular focus of this paper and therefore should be regarded as supplementary to the present hypothesis. Furthermore, N-acetylglucosamine also participates in additional glycosylation pathways affecting protein function, immune signaling, and inflammation (28). Given these systemic functions, reductions in either the availability or utilization of N-acetylglucosamine likely have broad effects across many different tissues (29). Although complex, glycosylation processes generally depend upon a small number of core substrates, including N-acetylglucosamine (11), thereby providing a reasonable mechanistic basis for how decreased availability or utilization of N-acetylglucosamine could simultaneously degrade cartilage and alter endothelial glycocalyx structure.

Therefore, if this hypothesis proves valid, then OA could serve as a clinically feasible indicator of an individual's potential vascular vulnerability. Additionally, interventions aimed at modulating N-acetylglucosamine-related pathways may provide novel strategies for managing both OA and CVD.

### Translational implications

Lack of N-acetylglucosamine may be overcome by exercise, diet and supplementation. Regular exercise correlates with a reduction in insulin resistance (30). Conversely, in patients with knee OA, lack of regular exercise results in an increased risk of cardiovascular events (6). Diet can be a rich source of N-acetylglucosamine, including from whole foods such as mushrooms, in which the linear polymer of N-acetylglucosamine, chitin, can be converted to the monomer by human acidic chitinase found in the stomach (31).

Theoretically, supplementation has a role when the organism can't make sufficient N-acetylglucosamine via the hexosamine biosynthetic pathway, when the pathway is blocked or impaired, as in insulin resistance, to maintain health. Whether supplementation will be a benefit in conditions such as progressive multiple sclerosis (32), Alzheimer's, or Parkinson's (33) is yet to be determined. In progressive multiple sclerosis, an inflammatory disease, blood levels of N-acetylglucosamine and its epimer were lower than in normal subjects (32), and the administration of N-acetylglucosamine has been shown to reduce inflammatory cytokines in blood and to lower serum neurofilament light chain, a marker of neuronal injury (29). While these studies do not deal with blood vessels or cartilage, they show that low N-acetylglucosamine is associated with inflammation and that administration of N-acetylglucosamine may mitigate inflammation. The clinical availability of an N-acetylglucosamine blood test could help define which patients, irrespective of their clinical diagnoses, might benefit from N-acetylglucosamine supplementation.

### Limitations and alternative explanations

There are several limitations associated with this hypothesis. Firstly, the evidence supporting the hypothesis is subject to bias inherent in the study designs. The relationship observed in epidemiological research may be a reflection of common risk factors (e.g., obesity, sedentary lifestyle) rather than the purported mechanism. While physical inactivity is a known CVD risk factor, the risk observed in younger OA patients (diagnosed under 40 years old) suggests a biological vulnerability that may precede the effects of sedentary lifestyles (5). Likewise, observational studies that investigate supplementation use are also subject to confounding, including healthy-user bias. Importantly, neither of these study designs is conducive to uncovering causal relationships. Moreover, specific details of how the hypothesized mechanism unfolds remain unclear.

Potential alternative explanations for the observed relationship include systemic inflammation, biomechanical limitation resulting in reduced levels of physical activity, and metabolic dysregulation independent of the hypothesized mechanism. Thus, future studies should be designed so that temporal associations, confounding variables, and mechanistic specificity can be determined.

We recognize that N-acetylglucosamine deficiency may be one factor that impacts blood vessels and is not the only factor. Intracellular hyperglycemia promotes the production of mitochondrial reactive oxygen species and advanced glycation products, which are accepted causes of vascular dysfunction (34). As the field progresses, we expect that the two different perspectives will merge. For example, in an ischemic mouse heart model, loss of N-acetylglucosamine attached to serine (O-GlcNAc) was associated with the loss of mitochondrial membrane potential, and increasing O-GlcNAc levels attenuated this loss (35). Mammalian biology is complex. No one pathway can explain it all. Our hypothesis is but one arm. We contend that it is worth further exploration.

## Conclusion

Shared dependence upon N-acetylglucosamine-derived glycans offers one possible mechanistic linkage between OA and CVD. Clarification of this relationship may have implications for both cardiovascular risk assessment and therapeutic development.

## Data Availability

The original contributions presented in the study are included in the article/Supplementary Material, further inquiries can be directed to the corresponding author.
